# Global changing trends in incidence and mortality of gastric cancer by age and sex, 1990-2019: Findings from Global Burden of Disease Study

**DOI:** 10.7150/jca.62734

**Published:** 2021-09-21

**Authors:** Tongchao Zhang, Hui Chen, Yuan Zhang, Xiaolin Yin, Jinyu Man, Xiaorong Yang, Ming Lu

**Affiliations:** 1 Clinical Epidemiology Unit, Qilu Hospital of Shandong University, Jinan, China.; 2 Clinical Research Center of Shandong University, Cheeloo College of Medicine, Shandong University, Jinan, China.; 3 Department of Epidemiology and Health Statistics, School of Public Health, Cheeloo College of Medicine, Shandong University, Jinan, China.; 4 Department of Gastroenterology, Qilu Hospital, Cheeloo College of Medicine, Shandong University, Jinan, China.

**Keywords:** Gastric cancer, Epidemiology, Global, Incidence, Mortality, Temporal trend

## Abstract

**Background:** The global disease burden of gastric cancer (GC) is still heavy. Understanding the patterns and trends of the global GC burden is important for developing precise prevention strategies.

**Materials and Methods:** The data of GC burden were retrieved from the Global Burden of Disease Study (2019). The estimated annual percentage change (EAPC) was calculated to estimate the temporal trends of the age-standardized incidence and mortality rates (ASIR and ASMR) of global GC by age-specific groups (15-49, 50-69, and ≥70 years), sexes, socio-demographic indexes (SDIs), regions, and countries.

**Results:** In 2019, the ASIR and ASMR of global GC increased with age in both sexes, and reached a peak in the older 70 age group. The ASIR and ASMR in males were higher than those in females. From 1990 to 2019, the global number of GC incident cases increased in both sexes in all age-specific groups; while the ASIR of GC decreased, and the most significant decrease was observed in the 50-69 age group [males: EAPC=-1.34, 95% CI: (-1.49, -1.18); females: EAPC= -2.09, 95% CI: (-2.22, -1.96)]. During the study period, downward trends in ASIR of GC were observed in both sexes in most SDI regions, GBD regions, and countries. Similar trends in ASMR of GC were also observed.

**Conclusion:** The global GC incidence and mortality rates decreased from 1990 to 2019 in both sexes, most GBD regions, and most countries. However, the GC burden was still heavy in some GBD regions and countries in special age-specific groups. It is important to formulate and implement tertiary prevention strategies based on the GC burden of age-specific groups in different regions and countries.

## Introduction

During the past few decades, both rates of incidence and mortality of gastric cancer (GC) were declined 1, 2 due to the increased understanding of the epidemiology, molecular mechanisms, and clinical diagnosis and treatment 3. However, GC is still the fifth most common cancer and the third leading cause of cancer death in the world 1. Since the symptoms of most patients with early GC are not obvious and specific, they are often diagnosed in the advanced stages 3, 4. Despite the improvement of early detection and treatment strategies, the overall 5-year survival rate of GC patients remains poor in most countries of the world 4-7.

The epidemiology of GC presents marked geographical variations in the world 7. The GC incidence rates are high in Eastern Asia, Latin America, and Eastern Europe, while being lower in Northern America, Northern Europe, and African 1, 3, 6-8. The GC mortality rates between different regions are often closely related to the incidence rates and human development index (HDI) 2. Meanwhile, the incidence and temporal trends of GC vary by age and sex 5, 7, 9. The incidence rate of GC in males is about 2 times higher than in females 1, 5, 9. During the past few decades, a downward trend of GC burden has been observed among the elderly population (>50 years old), while these trends are not obvious among youngers 10, 11.

Many studies have evaluated the GC burden at the global, regional, and national levels 2, 7, 9, 12, however, the GC burden of different sexes and age-specific groups remains unknown. In this study, based on the latest database of the Global Burden of Diseases (GBD) study (2019), we comprehensively evaluated the global changing trends of GC incidence and mortality. Our results enriched the research content of the global disease burden of GC, and could also provide an important basis for the precise prevention of GC in people of different age-specific groups, sexes, SDIs, regions, and countries.

## Materials and methods

### Study data sources

The detailed methods of data acquisition and modeling estimations of the GBD study have been reported previously 7, 13-16. Annual data of global GC burden were retrieved from the Institute for Health Metrics and Evaluation (IHME, query tool: http://ghdx.healthdata.org/gbd-results-tool) on the GBD study. The GBD study (2019) geographically grouped 204 countries into 21 regions and nested them in 7 super regions 16. According to quintiles of the socio-demographic index (SDI, a comprehensive indicator including fertility rate, education level, and income), these countries were also stratified into 5 regions: low, low-middle, middle, high-middle, and high 7, 16. Here, we collected the data based on the following criteria: First, for location, we selected the options of the global, SDI regions, GBD regions, and countries. Second, the year was from 1990 to 2019, and we chose “incidence” and “death” for measures. Since the lack of GC cases among people aged under 15 years, we used the data from people aged over 15 years.

### Estimation of incidence and mortality

The GC incidence and mortality in the GBD dataset were estimated generally in the following steps: (1) the mortality-to-incidence ratio (MIR) was computed by using the data sources that reported incidence and death of GC with international disease classification codes 7, 16, 17. (2) multiplying cancer register incidence data by the MIR to calculate the mortality estimates 7, 16, 17; (3) all these data were used as input to determine the cancer-specific mortality of GC through the Cause of Death Ensemble model process 7, 16, 17; and (4) the incidence was generated by dividing the cancer-specific mortality of GC estimates by the MIR 7, 16, 17. The age-standardized incidence and mortality rates (ASIR and ASMR) of GC were estimated using the WHO World Standard Population Distribution 2001.

### Statistical analysis

We described the numbers and age-standardized rates of GC incidence and mortality by grouping the data into 6 subgroups by sex and age-specific groups (15-49, 50-69, and ≥70 years). We also used the indicator of the estimated annual percentage change (EAPC) to describe the temporal trend of ASIR and ASMR of GC from 1990 to 2019. Based on a regression model fitted to the natural logarithm of the rate [ln(rate) = α + β*(calendar year) + ε], EAPC was calculated [100 × (exp(β)-1)] 17-19. The 95% confidence interval (CI) of EAPC was also obtained from the fitted model 17-19. All results were visualized using the packages of ggplot2 and RcolorBrewer of the R program (Version 3.6.2; R Foundation for Statistical Computing, Vienna, Austria).

### Ethics

The GBD study is a publicly available database, all participants' data were anonymous. Therefore, the study is exempt from ethical review.

## Results

### Global incidence changing trends in GC by sex in different age-specific groups

**Figure 1:**
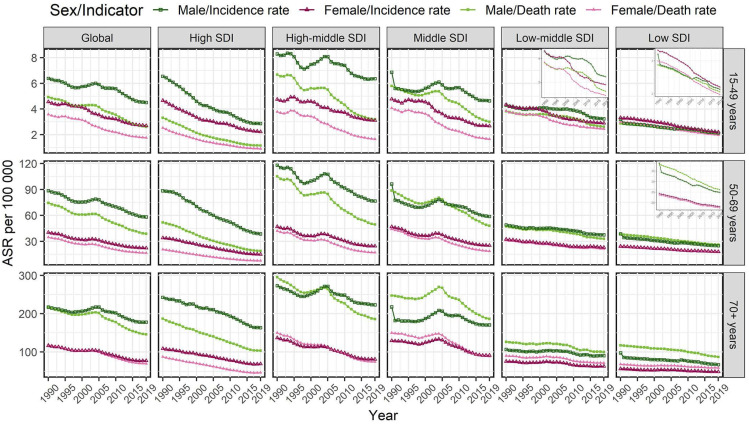
Temporal trends in the age-standardized incidence and mortality rates of gastric cancer in age-specific groups by sex and sociodemographic index (SDI) region from 1990 to 2019.

In all age-specific groups, the global GC number of incident cases increased from 1990 to 2019 in both sexes, while the ASIR of GC decreased in males [EAPC= -1.07, 95% CI: (-1.26, -0.88) for the 15-49 age group; EAPC= -1.34, 95% CI: (-1.49, -1.18) for the 50-69 age group; EAPC= -0.62, 95% CI: (-0.78, -0.46) for the older 70 age group] and females [EAPC= -2.00, 95% CI: (-2.13, -1.88) for the 15-49 age group; EAPC= -2.09, 95% CI: (-2.22, -1.96) for the 50-69 age group; EAPC= -1.49, 95% CI: (-1.64, -1.34) for the older 70 age group], particularly in females (Tables 1 and 2, Figure 1). Meanwhile, the ASIR of global GC increased with age in both sexes, and reached a peak in the older 70 age group in 2019 (Tables 1 and 2).

In 2019, the highest ASIR of GC was seen in males in the high-middle-SDI region in all age-specific groups, while the highest ASIR of GC was seen in females in the high-middle-SDI region aged 15-49 years old and the middle-SDI region aged 50-69 and over 70 years old (Tables 1 and 2). The ASIR of GC decreased from 1990 to 2019 in most SDI regions in both sexes in all age-specific groups, particularly in the high-SDI region (Tables 1 and 2, Figure 1). Moreover, during this period, the ASIR of GC in the 50-69 age group decreased more than that in the other two age-specific age groups in most SDI regions (Tables 1 and 2, Figure 1).

**Figure 2:**
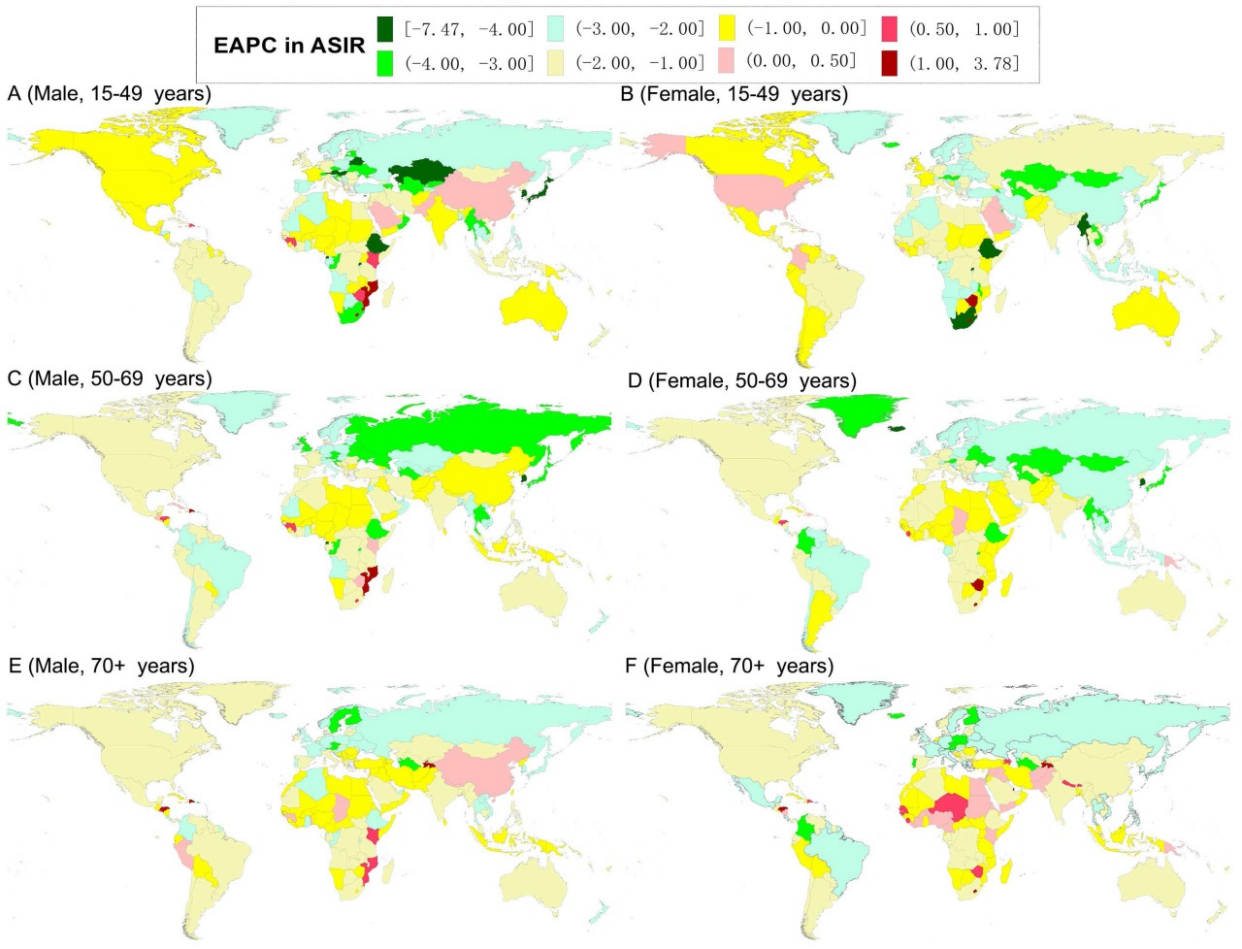
The world map of estimated average percentage change (EAPC) in the age-standardized incidence rates of gastric cancer by sex and age-specific groups from 1990 to 2019.

In 2019, the highest ASIR of GC was observed in males in East Asia (for the 15-49 age group) and North Africa and Middle East (for the 50-69 and older 70 age groups), and in females in South Asia (for the 15-49 and 50-69 age groups) and North Africa and Middle East (for the older 70 age group) (Tables 1 and 2). Meanwhile, the highest ASIR of GC was observed in males in the Solomon Islands (for the 15-49 age group), Georgia (for the 50-69 age group), and Mongolia (for the older 70 age group); while in females in Afghanistan (for the 15-49 age group), Mongolia (for the 50-69 age group), and Bolivia (for the older 70 age group) (Tables S1 and S2). The ASIR of GC decreased from 1990 to 2019 in both sexes in most GBD regions and countries in all age-specific groups (Tables 1, 2, S1, and S2; Figure 2). For the ASIR, the regions with the most significant decreases were in males in the High-income Asia Pacific (for the 15-49 and 50-69 age groups) and Eastern Europe (for the older 70 age group) and females in High-income Asia Pacific (for the all age-specific groups) (Tables 1 and 2). The countries with the most significant decline were in males in Equatorial Guinea (for the 15-49 age group), Singapore (for the 50-69 age group), and Austria (for the older 70 age group), and in females in the Maldives (for the 15-49 and 50-69 age groups) and Trinidad and Tobago (for the older 70 age group) (Tables S1 and S2, Figure 2). However, the ASIR increased in males in East Asia (especially in China in the 15-49 and older 70 age groups), and in females in High-income North America for the 15-49 age group and Western Sub-Saharan Africa for the older 70 age group (Tables 1, 2, S1, and S2; Figure 2).

### Global mortality changing trends in GC by sex in different age-specific groups

In 2019, in all age-specific groups, the global GC ASMR was higher in males than in females, and also reached a peak in the older 70 age group. During the study period, the ASMR of GC also decreased in both sexes [males: EAPC= -2.16, 95% CI: (-2.40, -1.92) for the 15-49 age group; EAPC= -2.18, 95% CI: (-2.36, -2.00) for the 50-69 age group; EAPC= -1.30, 95% CI: (-1.50, -1.11) for the older 70 age group; females: EAPC= -2.74, 95% CI: (-2.91, -2.56) for the 15-49 age group; EAPC= -2.64, 95% CI: (-2.79, -2.50) for the 50-69 age group; EAPC= -1.91, 95% CI: (-2.08, -1.74) for the older 70 age group], especially in the 15-49 and 50-69 age groups (Tables 3 and 4, Figure 1).

In 2019, the highest ASMR of GC was seen in males in the high-middle-SDI region in the 15-49 and 50-69 age groups and middle-SDI region in the older 70 age group, and in females in the middle-SDI region in all age-specific groups (Tables 3 and 4). In all age-specific groups, downward trends of ASMR of GC were observed in all SDI regions in both sexes (Tables 3 and 4, Figure 1).

**Figure 3:**
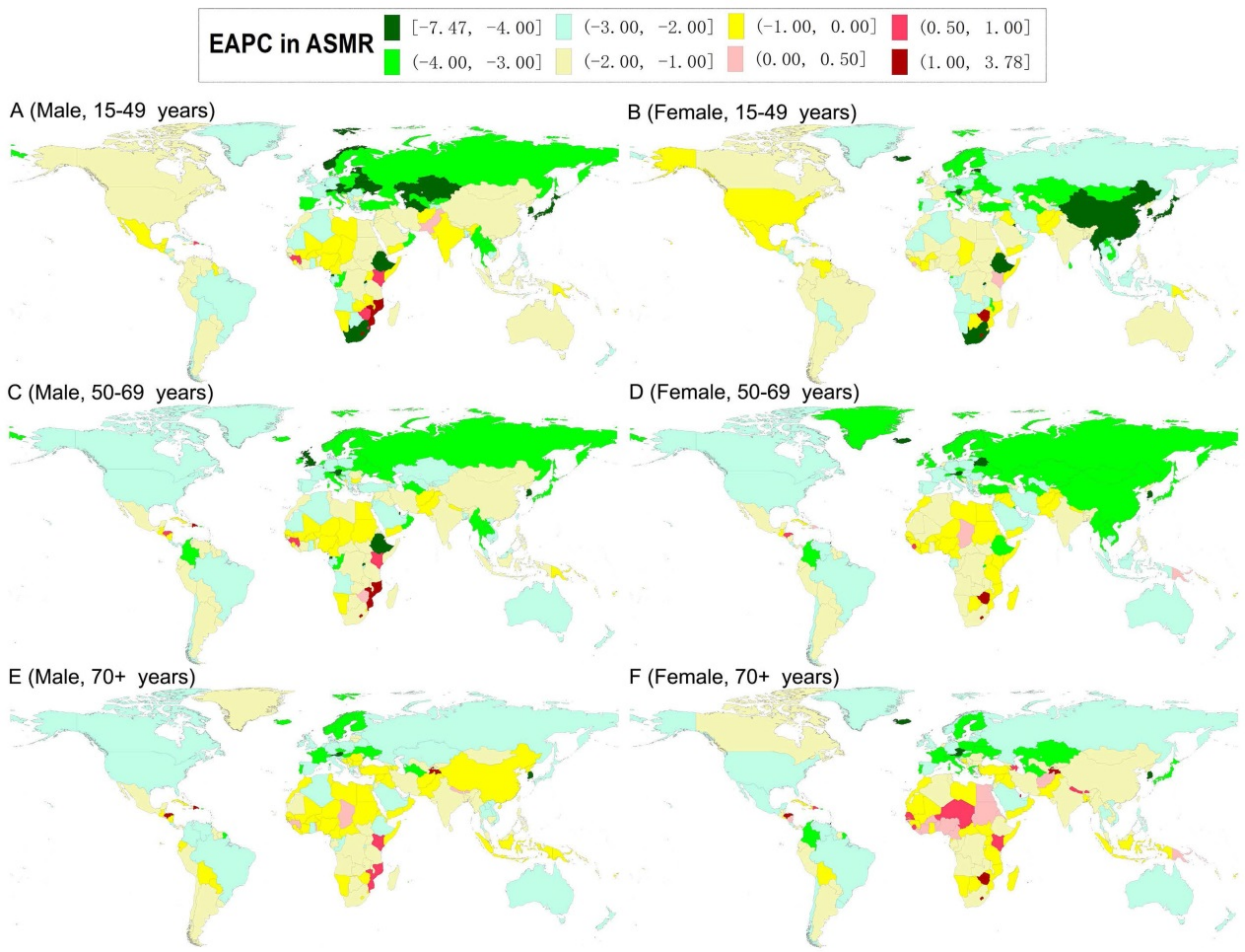
The world map of estimated average percentage change (EAPC) in the age-standardized mortality rates of gastric cancer by sex and age-specific groups from 1990 to 2019.

In 2019, the highest ASMR of GC was observed in males in South Asia (for the 15-49 age group) and North Africa and Middle East (for the 50-69 and older 70 age groups), and in females in South Asia (for the 15-49 and 50-69 age groups) and North Africa and Middle East (for the older 70 age group) (Tables 3 and 4). At the national level, the highest ASMR of GC was observed in males in the Solomon Islands (for the 15-49 age group) and Mongolia (for the 50-69 and older 70 age groups); while in females in Afghanistan (for the 15-49 age group), Mongolia (for the 50-69 age group), and Bolivia (for the older 70 age group) (Tables S3 and S4). During the study period, downward trends of ASMR of GC were also found in most GBD regions and countries in both sexes in all age-specific groups (Tables 3, 4, S3, and S4; Figures 3). For the ASMR, the most significant decrease was observed in males in the High-income Asia Pacific (for the 15-49 and 50-69 age groups) and Western Europe (for the older 70 age group) and females in High-income Asia Pacific (for the all age-specific groups) (Tables 3 and 4). At the national level, the most pronounced decrease was found in South Korea (for the 15-49 age group) and Singapore (for the 50-69 and older 70 age groups), and females in the Maldives (for the 15-49 age group) and South Korea (for the 50-69 and older 70 age groups) (Tables S3 and S4, Figure 3).

## Discussion

Globally, GC remains an important health problem despite the incidence rate has declined during the past few decades 7, 9, 20. Using the latest GC burden data in the GBD study, we fully evaluated the global burden of GC by age, sex, and different regions and counties. Consistent with the previous results 7, 9, 20-22, in our study, a downward trend in the global incidence and mortality rates of GC were observed in both sexes in all age-specific groups during the study period, while the numbers of GC incident cases increased accordingly. Meanwhile, the GC burden showed differences in age, sex, SDI regions, GBD regions, and countries.

Many reasons can explain the above results. Firstly, changes in the age structure of the population and the increase in population numbers in many regions and countries (such as East Asia) might lead to an increase in the number of incident cases 7, 23. Meanwhile, similar to the previous results 24, the GC burden increased with age, and the heaviest GC burden was observed in the older 70 age group in both sexes. GC is frequently diagnosed at 50-70 years old 10, 24, 25, which might result in a heavy GC burden in the elderly population. Meanwhile, the elderly population had more opportunities to be exposed to risk factors and have longer exposure time, this cumulative effect could lead to the accretion of mutations, which induced the occurrence of cancer 25, 26. Furthermore, the effect of the birth cohort is also an important reason, that is, the infection rates of *Helicobacter pylori (H. pylori)* have been decreased 20 and the lifestyles 9 have been changed among younger generations.

Secondly, in the high incidence of GC regions (such as Japan and South Korea), primary and secondary prevention strategies have been implemented 20. The control of risk factors is an important aspect, and the most noteworthy of which is *H. pylori*. *H. pylori* is classified as a group I human carcinogen 27, about 4.4 billion people (about 50% of the world's population) were infected with* H. pylori* around the world in 2015 28. Eradication of *H. pylori* could effectively reduce the GC incidence by about 50% 29-31. During the past few decades, the prevalence of *H. pylori* decreased in highly developed countries while remained stable in developing and newly industrialized countries 28, 32, which might contribute to the overall decline in the GC incidence rate. Meanwhile, the identification and control of other potentially modifiable risk factors may also contribute to the decline of the GC burden, such as smoking, alcohol drinking, the change of food preservation, chemical or radiation exposure, obesity, poor oral hygiene habits, insufficient intake of vegetables and fruits, and a high-sodium diet 9, 21, 22, 33-35.

Another important aspect is the successful implementation of population-based screening programs for middle-aged adults in some high GC burden countries (>50 years old in Japan and >40 years old in South Korea) 2, 36. In our study, the most significant decrease in incidence and mortality rates of GC was seen in the 50-69 age group in most regions and countries in both sexes. Therefore, the implementation of these strategies would have two effects. On the one hand, the incidence of GC would decrease first, which leads to a reduction in mortality of GC, and on the other hand, the early diagnosis and treatment of GC could significantly improve survival and subsequently decrease the mortality of GC, which could be illustrated partly by the difference between incidence trend and mortality trend, especially in higher SDI regions. Notably, East Asia (especially in China) carries most of the world's number of GC incident cases and deaths in 2019, however, with the implementation of various strategies, a further decrease could be observed.

Thirdly, the improvement of socioeconomic status 7, 8, 20, 24 and the advancement of medical technology and treatment 3 might contribute to a decline in the GC burden and also cause differences in the changing trends of GC burden in different regions and countries. Moreover, corresponds to other research reports 2, 9, 12, the GC burden in males was much higher than that in females, and this difference increased with age and reached a peak in the 50-69 age group. Males first have more opportunities to be exposed to occupational and environmental risk factors 5, 22. Meanwhile, the differences in sex-steroid hormones between males and females may also be a reason 5, 9. The estrogens were found to protect against the development of GC 37, 38. The incidence of GC in postmenopausal females was similar to that of males, however, it could lag by 10-15 years later 5, 38.

There were some limitations in our study. Firstly, the GC can be anatomically divided GC into cardia gastric cancer (CGC) and non-cardia gastric cancer (NCGC) 20. The temporal trend of the incidence and risk factors for these two subtypes of GC are different 5, 24, 39. In contrast to the decreases in NCGC incidence rate, the incidence rate of CGC recently increased (especially among younger) 2, 24. However, we could not evaluate the disease burden of GC from an anatomical perspective. Secondly, except for a high-sodium diet and smoking 7, other important risk factors for GC were not included in the GBD database. Therefore, in this study, we did not evaluate the burden of GC attributable to risk factors. Thirdly, because of the differences in data sources and estimation methods in the GBD study 7, our estimates might be higher than in other studies. However, our main findings generally agree with other studies. Fourthly, the data of the GBD study were collected from multiple sites and systems, we should not ignore the limitations of missing reports. Lastly, during the past three decades, the proportion of the population covered by these systems has changed. Therefore, it should be cautious when interpreting our results.

In conclusion, our study demonstrated that the global GC incidence and mortality rates decreased from 1990 to 2019, in both sexes, all age-specific groups, and most GBD regions and countries. However, the burden of GC was still heavy in some regions countries. To reduce the burden of GC, it is important to formulate and implement tertiary prevention strategies based on the GC burden of age-specific groups in different regions and countries.

## Supplementary Material

Supplementary tables.

## Figures and Tables

**Table 1:** Incident cases and age-standardized incidence rates of gastric cancer in 1990 and 2019 and the estimated annual percentage changes from 1990 to 2019 among males by age, SDI, and region

ASIR-Male Characteristics	15-49 years					50-69 years					≥70 years				
	1990		2019		1990-2019	1990		2019		1990-2019	1990		2019		1990-2019
	N*10^3^	ASIR/10^5^	N*10^3^	ASIR/10^5^	EAPC in ASIR (%, 95% CI)	N*10^3^	ASIR/10^5^	N*10^3^	ASIR/10^5^	EAPC in ASIR (%, 95% CI)	N*10^3^	ASIR/10^5^	N*10^3^	ASIR/10^5^	EAPC in ASIR (%, 95% CI)
Global	73.55	6.38	90.81	4.51	-1.07 (-1.26,-0.88)	297.39	88.51	395.43	58.17	-1.34 (-1.49,-1.18)	177.20	217.20	360.64	177.92	-0.62(-0.78, -0.46)
**SDI**															
Low SDI	2.80	3.20	4.40	2.13	-1.10 (-1.19, -1.02)	8.65	38.66	11.24	24.86	-1.24 (-1.33, -1.15)	4.56	97.67	7.02	67.07	-0.86 (-0.98, -0.74)
Low-middle SDI	9.35	4.29	13.65	3.23	-0.80 (-0.98, -0.61)	27.50	49.02	43.56	37.57	-0.85 (-0.95, -0.74)	12.83	106.71	28.05	90.42	-0.54 (-0.67, -0.42)
Middle SDI	24.71	6.86	31.07	4.64	-0.66 (-0.94, -0.37)	90.58	96.31	130.44	58.84	-0.87 (-1.15, -0.59)	43.47	217.71	96.17	170.62	-0.24 (-0.50, 0.02)
High-middle SDI	22.21	8.29	27.49	6.36	-0.89 (-1.10, -0.68)	103.76	118.09	127.33	76.64	-1.38 (-1.60, -1.17)	56.32	273.17	109.72	223.22	-0.57 (-0.74, -0.40)
High SDI	14.46	6.55	8.14	2.86	-2.99 (-3.10, -2.87)	66.83	88.44	50.72	38.58	-3.08 (-3.19, -2.98)	59.96	242.71	93.47	163.58	-1.40 (-1.50, -1.30)
**Region**															
High-income Asia Pacific	9.58	19.15	3.44	6.24	-4.23 (-4.39, -4.07)	40.72	252.61	25.15	94.30	-3.50 (-3.63, -3.37)	29.47	684.71	55.56	380.62	-2.10 (-2.20, -2.00)
Central Asia	1.41	11.67	1.22	5.28	-3.25 (-3.50, -3.01)	5.26	139.94	4.70	71.01	-2.23 (-2.38, -2.07)	1.65	233.85	1.78	169.50	-0.87 (-1.04, -0.71)
East Asia	30.55	10.67	49.75	10.74	0.41 (0.04, 0.78)	122.79	148.98	229.84	120.92	-0.27 (-0.65, 0.10)	59.55	355.16	180.69	361.95	0.38 (0.06, 0.71)
South Asia	6.81	13.03	9.96	9.66	-0.93 (-1.07, -0.78)	17.34	135.41	25.58	90.25	-1.39 (-1.50, -1.28)	7.73	310.99	15.33	215.12	-1.51 (-1.75, -1.27)
Southeast Asia	2.96	3.27	3.81	2.05	-1.92 (-2.06, -1.78)	8.98	40.51	13.16	24.33	-2.00 (-2.13, -1.87)	4.42	93.33	7.52	65.72	-1.45 (-1.55, -1.35)
Australasia	0.12	2.27	0.14	1.88	-0.49 (-0.61, -0.37)	0.63	34.82	0.77	22.03	-1.75 (-1.86, -1.64)	0.74	128.46	1.28	82.93	-1.69 (-1.79, -1.60)
Caribbean	0.22	3.03	0.30	2.54	-0.67 (-0.84, -0.49)	0.80	39.00	1.21	29.83	-0.78 (-0.95, -0.61)	0.78	111.59	1.12	79.61	-1.04 (-1.15, -0.93)
Central Europe	1.61	5.20	0.93	2.74	-2.45 (-2.60, -2.29)	9.24	76.82	6.62	42.57	-2.07 (-2.13, -2.01)	5.85	195.18	6.36	118.08	-1.71 (-1.77, -1.65)
Eastern Europe	6.34	12.37	3.60	6.22	-3.14 (-3.50, -2.77)	32.59	162.21	18.77	75.93	-3.07 (-3.31, -2.82)	11.02	276.65	9.60	152.88	-2.25 (-2.38, -2.11)
Western Europe	3.77	3.75	2.79	2.37	-1.69 (-1.80, -1.59)	23.18	56.03	17.11	29.55	-2.28 (-2.38, -2.17)	27.53	198.86	31.85	111.57	-2.14 (-2.22, -2.07)
High-income North America	1.57	2.13	1.65	1.82	-0.58 (-0.65, -0.51)	7.07	29.36	9.41	20.13	-1.48 (-1.59, -1.37)	9.52	106.84	12.31	69.04	-1.79 (-1.89, -1.69)
Andean Latin America	0.50	6.96	0.71	4.56	-1.52 (-1.71, -1.33)	1.52	89.44	2.43	54.36	-1.58 (-1.68, -1.47)	1.31	269.48	3.76	248.53	-0.16 (-0.26, -0.07)
Central Latin America	1.38	4.51	2.42	3.94	-0.73 (-0.85, -0.61)	3.88	56.40	7.14	38.49	-1.59 (-1.70, -1.48)	3.27	170.80	7.05	119.68	-1.60 (-1.72, -1.47)
Southern Latin America	0.56	5.01	0.58	3.37	-1.33 (-1.44, -1.23)	2.71	74.93	2.99	48.72	-1.55 (-1.64, -1.45)	2.29	214.89	3.37	157.49	-1.07 (-1.15, -1.00)
Tropical Latin America	1.58	5.14	1.80	2.99	-1.90 (-2.04, -1.77)	5.58	73.27	7.64	39.45	-2.20 (-2.29, -2.12)	3.55	185.84	6.40	113.11	-1.75 (-1.85, -1.64)
North Africa and Middle East	2.34	16.49	3.93	10.23	-1.70 (-2.00, -1.40)	8.08	222.62	12.54	140.93	-1.48 (-1.73, -1.22)	4.43	521.60	10.06	454.65	-0.25 (-0.44, -0.05)
Oceania	0.09	6.65	0.20	6.55	0.03 (-0.04, 0.10)	0.14	47.50	0.30	43.43	-0.31 (-0.35, -0.26)	0.06	119.46	0.13	104.27	-0.54 (-0.60, -0.49)
Central Sub-Saharan Africa	0.31	3.75	0.54	2.37	-1.66 (-1.73, -1.58)	1.00	49.50	1.50	32.05	-1.63 (-1.72, -1.53)	0.42	106.02	0.57	76.51	-1.21 (-1.28, -1.13)
Eastern Sub-Saharan Africa	0.88	3.18	1.41	2.00	-1.82 (-1.92, -1.72)	2.64	39.22	3.47	24.89	-1.73 (-1.83, -1.62)	1.36	89.17	1.97	65.30	-1.18 (-1.28, -1.08)
Southern Sub-Saharan Africa	0.32	3.51	0.37	1.97	-2.67 (-3.08, -2.26)	0.69	32.57	1.09	24.92	-1.13 (-1.65, -0.62)	0.39	76.54	0.59	61.67	-1.11 (-1.51, -0.71)
Western Sub-Saharan Africa	0.67	2.04	1.27	1.64	-0.72 (-0.77, -0.67)	2.56	31.10	4.02	25.48	-0.51 (-0.61, -0.41)	1.87	106.24	3.34	90.14	-0.43 (-0.50, -0.36)

Abbreviations: ASIR: age-standardized incidence rate; CI: confidence interval; EAPC: estimated annual percentage change; N: The numbers of cases are at 1,000 scales, SDI: sociodemographic index.

**Table 2:** Incident cases and age-standardized incidence rates of gastric cancer in 1990 and 2019 and the estimated annual percentage changes from 1990 to 2019 among females by age, SDI, and region

ASIR-Female Characteristics	15-49 years					50-69 years					≥70 years				
	1990		2019		1990-2019	1990		2019		1990-2019	1990		2019		1990-2019
	N*10^3^	ASIR /10^5^	N*10^3^	ASIR /10^5^	EAPC in ASIR (%, 95% CI)	N*10^3^	ASIR/10^5^	N*10^3^	ASIR /10^5^	EAPC in ASIR (%, 95% CI)	N*10^3^	ASIR /10^5^	N*10^3^	ASIR /10^5^	EAPC in ASIR (%, 95% CI)
Global	52.42	4.56	53.52	2.70	-2.00 (-2.13, -1.88)	142.56	40.32	159.64	22.31	-2.09 (-2.22, -1.96)	140.28	116.68	209.78	77.31	-1.49 (-1.64, -1.34)
**SDI**															
Low SDI	3.04	3.32	4.70	2.20	-1.51 (-1.58, -1.45)	5.08	24.29	8.26	18.11	-1.06 (-1.10, -1.02)	2.65	56.43	5.70	49.04	-0.44 (-0.49, -0.38)
Low-middle SDI	9.38	4.33	12.41	2.90	-1.53 (-1.64, -1.41)	17.17	32.40	27.92	23.00	-1.22 (-1.31, -1.13)	9.57	75.65	22.97	62.64	-0.71 (-0.85, -0.57)
Middle SDI	16.99	4.77	17.66	2.67	-2.33 (-2.55, -2.10)	43.28	46.88	58.50	25.28	-2.07 (-2.28, -1.86)	32.38	129.53	62.91	91.40	-1.26 (-1.55, -0.97)
High-middle SDI	12.82	4.75	12.82	3.11	-1.70 (-1.86, -1.54)	48.04	47.07	44.54	24.59	-2.25 (-2.48, -2.03)	48.45	137.12	58.96	81.15	-1.85 (-2.00, -1.70)
High SDI	10.18	4.66	5.90	2.23	-2.50 (-2.61, -2.39)	28.96	34.05	20.37	15.06	-3.07 (-3.16, -2.99)	47.18	108.88	59.16	69.06	-1.74 (-1.81, -1.67)
**Region**															
High-income Asia Pacific	7.11	14.65	2.97	5.89	-3.23 (-3.35, -3.11)	16.87	91.98	8.96	33.62	-3.65 (-3.73, -3.57)	19.98	282.95	32.08	135.59	-2.78 (-2.85, -2.70)
Central Asia	0.76	5.73	0.75	3.14	-2.39 (-2.56, -2.23)	2.57	53.84	2.31	28.90	-2.26 (-2.37, -2.16)	1.76	116.44	1.36	76.51	-1.27 (-1.45, -1.09)
East Asia	18.75	6.84	18.64	4.30	-1.99 (-2.27, -1.71)	53.41	67.86	69.22	35.98	-2.05 (-2.43, -1.67)	40.66	182.21	78.35	127.12	-1.14 (-1.55, -0.74)
South Asia	7.77	15.64	11.17	10.95	-1.27 (-1.37, -1.17)	12.36	106.93	22.35	77.67	-1.17 (-1.36, -0.98)	5.57	227.70	15.01	185.22	-1.06 (-1.34, -0.79)
Southeast Asia	2.74	2.81	2.54	1.38	-2.61 (-2.71, -2.50)	5.47	22.64	6.81	11.58	-2.52 (-2.61, -2.44)	3.50	57.31	6.22	38.15	-1.60 (-1.68, -1.52)
Australasia	0.09	1.63	0.10	1.30	-0.60 (-0.69, -0.51)	0.27	14.26	0.34	9.26	-1.65 (-1.75, -1.55)	0.53	58.56	0.82	41.21	-1.36 (-1.45, -1.26)
Caribbean	0.19	2.39	0.24	1.94	-0.72 (-0.81, -0.63)	0.43	20.11	0.67	15.33	-0.85 (-0.92, -0.78)	0.50	63.03	0.82	46.46	-0.93 (-1.01, -0.84)
Central Europe	0.91	2.90	0.53	1.66	-2.08 (-2.17, -1.99)	3.99	27.44	2.63	15.22	-1.98 (-2.03, -1.93)	4.86	95.94	4.65	51.49	-2.27 (-2.34, -2.20)
Eastern Europe	3.61	6.50	2.45	4.08	-2.02 (-2.30, -1.73)	17.99	59.60	9.61	28.50	-2.89 (-3.07, -2.70)	15.49	135.74	10.08	70.70	-2.49 (-2.60, -2.38)
Western Europe	2.37	2.40	1.82	1.58	-1.52 (-1.63, -1.41)	10.87	22.79	8.12	13.36	-1.86 (-1.95, -1.77)	26.23	101.78	24.77	57.97	-2.13 (-2.23, -2.03)
High-income North America	0.92	1.22	1.14	1.25	0.18 (0.08, 0.28)	3.33	12.05	4.29	8.59	-1.38 (-1.48, -1.29)	7.91	52.10	8.79	35.43	-1.57 (-1.66, -1.48)
Andean Latin America	0.48	6.40	0.80	5.00	-1.00 (-1.20, -0.79)	1.05	60.27	1.85	38.93	-1.68 (-1.81, -1.55)	1.12	205.03	2.83	162.85	-0.98 (-1.14, -0.81)
Central Latin America	1.21	3.75	2.29	3.43	-0.34 (-0.40, -0.27)	2.77	38.11	5.27	24.97	-1.72 (-1.83, -1.60)	2.99	138.31	6.34	85.28	-2.00 (-2.11, -1.88)
Southern Latin America	0.29	2.47	0.34	1.88	-0.90 (-0.97, -0.82)	1.03	24.91	1.20	17.39	-1.16 (-1.24, -1.08)	1.58	100.32	2.22	66.72	-1.47 (-1.58, -1.35)
Tropical Latin America	0.92	2.77	1.26	1.99	-1.05 (-1.15, -0.94)	2.21	26.57	3.32	15.01	-2.04 (-2.12, -1.97)	2.28	96.08	4.12	51.43	-2.26 (-2.34, -2.18)
North Africa and Middle East	2.10	14.68	3.34	9.45	-1.48 (-1.68, -1.28)	3.94	113.35	6.37	74.58	-1.44 (-1.62, -1.26)	2.59	321.39	6.02	296.95	0.02 (-0.34, 0.38)
Oceania	0.04	3.21	0.09	3.09	0.00 (-0.09, 0.09)	0.06	20.85	0.12	19.90	-0.13 (-0.16, -0.09)	0.04	70.46	0.09	69.65	-0.09 (-0.13, -0.05)
Central Sub-Saharan Africa	0.24	2.53	0.36	1.50	-1.87 (-1.96, -1.78)	0.54	22.91	0.81	15.48	-1.33 (-1.41, -1.26)	0.22	53.83	0.47	39.96	-1.05 (-1.08, -1.02)
Eastern Sub-Saharan Africa	1.03	3.29	1.30	1.69	-2.70 (-2.87, -2.53)	1.55	23.06	2.12	14.54	-1.80 (-1.89, -1.71)	0.76	48.44	1.49	40.47	-0.65 (-0.68, -0.62)
Southern Sub-Saharan Africa	0.24	2.26	0.25	1.27	-1.57 (-1.99, -1.16)	0.37	14.37	0.65	11.63	-0.36 (-0.65, -0.07)	0.40	49.32	0.66	39.31	-0.91 (-1.24, -0.58)
Western Sub-Saharan Africa	0.65	2.06	1.12	1.31	-1.52 (-1.59, -1.45)	1.47	20.49	2.63	15.13	-0.85 (-0.95, -0.76)	1.30	62.02	2.58	64.15	0.55 (0.36, 0.73)

Abbreviations: ASIR: age-standardized incidence rate; CI: confidence interval; EAPC: estimated annual percentage change; N: The numbers of cases are at 1,000 scales, SDI: sociodemographic index.

**Table 3:** Death cases and age-standardized mortality rates of gastric cancer in 1990 and 2019 and the estimated annual percentage changes from 1990 to 2019 among males by age, SDI, and region

ASMR-Male Characteristics	15-49 years					50-69 years					≥70 years				
	1990		2019		1990-2019	1990		2019		1990-2019	1990		2019		1990-2019
	N*10^3^	ASMR/10^5^	N*10^3^	ASMR/10^5^	EAPC in ASMR (%, 95% CI)	N*10^3^	ASMR/10^5^	N*10^3^	ASMR/10^5^	EAPC in ASMR (%, 95% CI)	N*10^3^	ASMR/10^5^	N*10^3^	ASMR/10^5^	EAPC in ASMR (%, 95% CI)
Global	56.51	4.93	52.59	2.61	-2.16 (-2.40, -1.92)	250.07	74.47	263.87	38.79	-2.18 (-2.36, -2.00)	174.73	217.10	295.04	146.25	-1.30 (-1.50, -1.11)
**SDI**															
Low SDI	2.48	2.87	4.19	2.06	-1.16 (-1.21, -1.10)	8.55	38.30	11.82	26.21	-1.37 (-1.41, -1.33)	5.35	117.39	8.98	87.21	-0.99 (-1.07, -0.90)
Low-middle SDI	8.24	3.82	11.01	2.62	-1.17 (-1.37, -0.97)	26.43	47.22	38.21	32.99	-1.18 (-1.31, -1.05)	14.87	126.46	30.57	100.03	-0.82 (-0.97, -0.68)
Middle SDI	20.67	5.80	20.19	3.00	-2.09 (-2.44, -1.74)	83.38	88.83	106.41	48.06	-1.81 (-2.11, -1.51)	48.36	247.56	103.73	186.48	-0.85 (-1.17, -0.54)
High-middle SDI	17.79	6.68	13.87	3.19	-2.71 (-2.98, -2.44)	92.29	105.13	82.50	49.55	-2.60 (-2.84, -2.36)	60.17	295.53	91.11	186.11	-1.52 (-1.71, -1.33)
High SDI	7.30	3.31	3.31	1.15	-3.81 (-3.95, -3.68)	39.34	51.89	24.85	18.84	-3.72 (-3.79, -3.64)	45.91	187.04	60.56	103.61	-2.12 (-2.18, -2.06)
**Region**															
High-income Asia Pacific	4.21	8.43	1.11	1.97	-5.48 (-5.65, -5.32)	20.35	126.56	10.44	38.79	-4.21 (-4.31, -4.11)	19.22	454.51	32.62	217.31	-2.70 (-2.78, -2.62)
Central Asia	1.23	10.36	1.05	4.54	-3.36 (-3.60, -3.12)	4.97	133.12	4.35	65.97	-2.31 (-2.46, -2.17)	1.88	265.36	1.97	189.28	-0.94 (-1.09, -0.79)
East Asia	25.12	8.84	23.00	4.89	-1.77 (-2.23, -1.31)	111.54	135.48	138.51	72.80	-1.78 (-2.20, -1.35)	65.17	402.80	144.28	299.01	-0.75 (-1.12, -0.37)
South Asia	6.07	11.8	8.62	8.45	-1.04 (-1.19, -0.89)	16.82	132.87	24.23	86.22	-1.48 (-1.59, -1.36)	9.05	412.07	17.74	283.05	-1.54 (-1.79, -1.28)
Southeast Asia	2.55	2.86	2.95	1.59	-2.32 (-2.47, -2.17)	8.67	39.21	11.63	21.59	-2.29 (-2.41, -2.17)	5.18	110.87	8.37	73.92	-1.62 (-1.71, -1.52)
Australasia	0.07	1.26	0.06	0.76	-1.58 (-1.70, -1.46)	0.41	22.64	0.38	10.80	-2.74 (-2.90, -2.57)	0.59	104.53	0.82	51.61	-2.63 (-2.76, -2.49)
Caribbean	0.19	2.60	0.24	2.07	-0.86 (-1.07, -0.66)	0.75	36.39	1.06	26.01	-1.03 (-1.21, -0.84)	0.86	123.79	1.15	80.99	-1.33 (-1.44, -1.21)
Central Europe	1.38	4.47	0.71	2.07	-2.92 (-3.08, -2.76)	8.57	71.18	5.60	35.81	-2.43 (-2.49, -2.36)	6.48	216.78	6.42	119.16	-2.04 (-2.09, -1.98)
Eastern Europe	4.96	9.73	2.28	3.92	-4.08 (-4.47, -3.68)	28.60	143.16	14.30	57.66	-3.71 (-3.99, -3.43)	11.93	301.20	9.55	151.68	-2.63 (-2.78, -2.48)
Western Europe	2.42	2.40	1.29	1.08	-2.87 (-2.97, -2.77)	17.28	41.65	10.08	17.34	-3.09 (-3.21, -2.96)	25.59	185.91	24.83	84.84	-2.85 (-2.93, -2.77)
High-income North America	0.84	1.14	0.69	0.76	-1.43 (-1.53, -1.32)	4.47	18.53	4.73	10.09	-2.26 (-2.44, -2.08)	7.07	79.66	7.80	42.78	-2.39 (-2.53, -2.25)
Andean Latin America	0.44	6.26	0.61	3.96	-1.66 (-1.85, -1.46)	1.49	87.85	2.26	50.75	-1.75 (-1.85, -1.64)	1.49	305.65	3.38	222.38	-0.99 (-1.09, -0.88)
Central Latin America	1.16	3.84	1.74	2.84	-1.31 (-1.43, -1.18)	3.64	53.01	6.04	32.62	-1.94 (-2.05, -1.84)	3.72	194.73	7.28	122.18	-1.96 (-2.07, -1.84)
Southern Latin America	0.46	4.14	0.41	2.36	-1.92 (-2.01, -1.82)	2.48	68.68	2.46	39.98	-1.95 (-2.04, -1.86)	2.55	242.12	3.43	159.79	-1.46 (-1.53, -1.38)
Tropical Latin America	1.37	4.51	1.43	2.37	-2.27 (-2.41, -2.14)	5.28	69.37	6.72	34.75	-2.46 (-2.54, -2.38)	4.02	214.20	6.80	119.81	-2.02 (-2.11, -1.93)
North Africa and Middle East	2.04	14.63	3.05	8.00	-2.12 (-2.41, -1.84)	7.74	215.24	10.94	124.33	-1.79 (-2.02, -1.55)	5.10	680.32	10.81	556.55	-0.47 (-0.65, -0.29)
Oceania	0.07	5.78	0.17	5.65	0.03 (-0.05, 0.10)	0.13	45.74	0.29	41.30	-0.34 (-0.39, -0.30)	0.07	142.17	0.15	123.27	-0.56 (-0.62, -0.50)
Central Sub-Saharan Africa	0.27	3.36	0.48	2.13	-1.64 (-1.72, -1.57)	1.00	49.62	1.47	31.62	-1.68 (-1.78, -1.59)	0.49	127.60	0.67	91.03	-1.25 (-1.32, -1.18)
Eastern Sub-Saharan Africa	0.78	2.84	1.26	1.81	-1.75 (-1.84, -1.65)	2.65	39.37	3.43	24.71	-1.76 (-1.86, -1.65)	1.59	106.57	2.32	78.08	-1.18 (-1.28, -1.08)
Southern Sub-Saharan Africa	0.29	3.14	0.32	1.72	-2.73 (-3.14, -2.33)	0.66	31.22	1.03	23.77	-1.14 (-1.68, -0.60)	0.46	90.61	0.68	72.49	-1.16 (-1.59, -0.73)
Western Sub-Saharan Africa	0.60	1.83	1.12	1.46	-0.73 (-0.78, -0.68)	2.54	30.89	3.92	24.95	-0.56 (-0.66, -0.45)	2.22	128.26	3.98	108.57	-0.43 (-0.50, -0.37)

Abbreviations: ASMR: age-standardized mortality rate; CI: confidence interval; EAPC: estimated annual percentage change; N: The numbers of cases are at 1,000 scales, SDI: sociodemographic index.

**Table 4:** Death cases and age-standardized mortality rates of gastric cancer in 1990 and 2019 and the estimated annual percentage changes from 1990 to 2019 among females by age, SDI, and region

ASMR-Female Characteristics	15-49 years					50-69 years					≥70 years				
	1990		2019		1990-2019	1990		2019		1990-2019	1990		2019		1990-2019
	N*10^3^	ASMR/10^5^	N*10^3^	ASMR/10^5^	EAPC in ASMR (%, 95% CI)	N*10^3^	ASMR/10^5^	N*10^3^	ASMR/10^5^	EAPC in ASMR (%, 95% CI)	N*10^3^	ASMR/10^5^	N*10^3^	ASMR/10^5^	EAPC in ASMR (%, 95% CI)
Global	40.88	3.58	34.74	1.75	-2.74 (-2.91, -2.56)	122.82	34.68	118.24	16.50	-2.64 (-2.79, -2.50)	143.32	119.22	192.70	69.71	-1.91 (-2.08, -1.74)
SDI															
Low SDI	2.70	3.00	4.16	1.97	-1.53 (-1.60, -1.47)	4.93	23.63	7.96	17.49	-1.08 (-1.12, -1.04)	3.12	67.82	6.76	58.62	-0.46 (-0.52, -0.40)
Low-middle SDI	8.22	3.85	10.40	2.44	-1.71 (-1.82, -1.59)	16.53	31.25	25.74	21.20	-1.36 (-1.44, -1.28)	11.18	90.00	26.03	71.30	-0.85 (-0.99, -0.70)
Middle SDI	14.28	4.06	10.90	1.65	-3.48 (-3.74, -3.21)	40.65	44.04	43.07	18.62	-2.93 (-3.16, -2.70)	37.01	149.92	60.96	88.35	-1.87 (-2.21, -1.53)
High-middle SDI	10.16	3.79	6.85	1.65	-3.27 (-3.53, -3.00)	43.01	42.02	31.05	17.05	-3.22 (-3.46, -2.98)	53.02	150.05	55.58	74.35	-2.50 (-2.67, -2.33)
High SDI	5.51	2.53	2.41	0.90	-3.59 (-3.72, -3.47)	17.66	20.62	10.38	7.63	-3.67 (-3.78, -3.57)	38.94	87.45	43.29	46.58	-2.36 (-2.43, -2.28)
**Region**															
High-income Asia Pacific	3.51	7.26	0.96	1.87	-4.89 (-5.01, -4.77)	8.65	47.11	3.70	13.74	-4.45 (-4.54, -4.36)	13.71	193.31	21.03	79.90	-3.35 (-3.46, -3.24)
Central Asia	0.65	4.98	0.63	2.64	-2.51 (-2.68, -2.35)	2.43	51.02	2.11	26.56	-2.38 (-2.48, -2.28)	2.02	132.65	1.52	85.08	-1.35 (-1.52, -1.18)
East Asia	15.54	5.73	9.21	2.09	-3.94 (-4.33, -3.54)	49.70	63.06	46.28	24.01	-3.24 (-3.66, -2.81)	46.03	209.89	71.72	115.68	-1.97 (-2.42, -1.52)
South Asia	6.84	14.04	9.69	9.60	-1.34 (-1.44, -1.24)	11.95	104.58	21.26	74.46	-1.24 (-1.42, -1.05)	6.51	300.67	17.53	244.03	-1.08 (-1.37, -0.79)
Southeast Asia	2.40	2.50	2.07	1.12	-2.90 (-3.01, -2.79)	5.28	21.85	6.10	10.41	-2.76 (-2.84, -2.68)	4.14	68.17	7.08	43.25	-1.76 (-1.83, -1.68)
Australasia	0.05	0.93	0.04	0.57	-1.51 (-1.59, -1.43)	0.18	9.81	0.18	4.94	-2.51 (-2.63, -2.38)	0.45	49.43	0.57	26.89	-2.22 (-2.36, -2.08)
Caribbean	0.16	2.04	0.19	1.59	-0.87 (-0.98, -0.76)	0.40	18.80	0.59	13.48	-1.09 (-1.17, -1.00)	0.58	72.06	0.88	48.87	-1.22 (-1.31, -1.12)
Central Europe	0.75	2.40	0.38	1.18	-2.66 (-2.77, -2.56)	3.67	25.16	2.17	12.43	-2.42 (-2.46, -2.37)	5.48	107.54	4.84	52.23	-2.61 (-2.68, -2.54)
Eastern Europe	2.69	4.86	1.43	2.36	-3.11 (-3.44, -2.77)	15.81	52.16	7.18	21.06	-3.60 (-3.81, -3.38)	16.93	147.29	10.27	69.58	-2.90 (-3.02, -2.77)
Western Europe	1.59	1.60	0.88	0.75	-2.72 (-2.81, -2.64)	8.29	17.29	4.80	7.87	-2.75 (-2.90, -2.60)	25.62	97.41	21.25	45.86	-2.78 (-2.90, -2.66)
High-income North America	0.53	0.70	0.54	0.58	-0.54 (-0.61, -0.46)	2.19	7.91	2.30	4.60	-2.06 (-2.21, -1.92)	6.30	40.29	6.26	23.44	-2.06 (-2.15, -1.97)
Andean Latin America	0.41	5.54	0.57	3.58	-1.66 (-1.87, -1.45)	1.02	58.72	1.72	36.23	-1.84 (-1.97, -1.71)	1.35	246.77	3.24	183.36	-1.21 (-1.39, -1.03)
Central Latin America	0.98	3.09	1.51	2.27	-1.09 (-1.17, -1.01)	2.53	34.90	4.16	19.72	-2.20 (-2.31, -2.08)	3.42	158.57	6.57	86.33	-2.38 (-2.47, -2.28)
Southern Latin America	0.24	2.03	0.24	1.32	-1.45 (-1.51, -1.38)	0.96	23.01	1.00	14.40	-1.54 (-1.61, -1.47)	1.83	115.74	2.42	70.48	-1.78 (-1.89, -1.67)
Tropical Latin America	0.78	2.39	0.98	1.55	-1.41 (-1.51, -1.31)	2.10	25.22	2.91	13.17	-2.32 (-2.39, -2.25)	2.65	113.01	4.61	56.13	-2.51 (-2.59, -2.44)
North Africa and Middle East	1.82	12.98	2.62	7.49	-1.84 (-2.03, -1.65)	3.78	109.68	5.60	66.28	-1.73 (-1.89, -1.57)	3.07	429.77	6.57	368.54	-0.22 (-0.59, 0.15)
Oceania	0.03	2.84	0.08	2.70	-0.02 (-0.11, 0.08)	0.05	20.21	0.12	19.04	-0.16 (-0.20, -0.12)	0.04	84.46	0.11	82.74	-0.11 (-0.15, -0.07)
Central Sub-Saharan Africa	0.21	2.28	0.32	1.35	-1.88 (-1.97, -1.79)	0.52	22.34	0.78	15.02	-1.35 (-1.42, -1.28)	0.26	64.51	0.56	47.90	-1.06 (-1.09, -1.03)
Eastern Sub-Saharan Africa	0.92	2.97	1.17	1.55	-2.62 (-2.78, -2.47)	1.51	22.43	2.10	14.45	-1.67 (-1.74, -1.60)	0.89	57.92	1.80	49.32	-0.55 (-0.57, -0.52)
Southern Sub-Saharan Africa	0.20	1.97	0.21	1.09	-1.51 (-1.91, -1.11)	0.35	13.68	0.62	10.96	-0.31 (-0.57, -0.05)	0.49	59.46	0.79	46.74	-0.93 (-1.23, -0.63)
Western Sub-Saharan Africa	0.58	1.87	1.00	1.18	-1.53 (-1.61, -1.46)	1.43	19.97	2.55	14.71	-0.86 (-0.96, -0.77)	1.55	74.71	3.09	77.22	0.55 (0.36, 0.73)

Abbreviations: ASMR: age-standardized mortality rate; CI: confidence interval; EAPC: estimated annual percentage change; N: The numbers of cases are at 1,000 scales, SDI: sociodemographic index.
